# Editorial: *In Vitro* models: opportunities and challenges in aquatic physiology

**DOI:** 10.3389/fphys.2023.1275350

**Published:** 2023-08-25

**Authors:** Francisco Javier Moyano, Hector Nolasco-Soria, Juan Fuentes

**Affiliations:** ^1^ Departamento Biología y Geología, University of Almeria, Almería, Spain; ^2^ Centro de Investigación Biológica del Noroeste (CIBNOR), La Paz, Baja California Sur, Mexico; ^3^ Center for Marine and Environmental Sciences (MARE), Lisbon, Portugal

**Keywords:** *in vitro*, fish, physiology, aquaculture, metabolism

Research on aquatic organisms is sometimes one step behind that performed on terrestrial species, with a good example of this being the still comparatively limited use of *in vitro* models adapted to simulate different aspects of the physiology of aquatic species. For practical reasons, most of these applications are focused on aquaculture species since they can be applied in the fields of nutrition, immunology, toxicology, and the general metabolism of economically valuable fish and crustaceans. Several reasons can explain such scarcity of research: on the one hand, the fact that modern aquaculture is a very recent activity compared with other types of animal husbandry which possess a much longer tradition of research based on the use of this type of model (i.e., ruminant nutrition); on the other hand, the great diversity of species presenting big differences in their anatomical, physiological, and biochemical features makes the development of standardized procedures difficult in an equivalent manner as those done for humans or terrestrial animals. In spite of this, great advances have taken place in recent years and *in vitro* models are increasingly used as routine techniques in different aspects of aquatic physiology. The works presented in the Research Topic are good examples of this, with studies developing models simulating different parts of the digestive tract of salmon or the intestinal microbiota of largemouth bass, as well as others using cell cultures of hepatocytes or intestinal cells in hybrid grouper and rainbow trout, respectively.

The paper by Radhakrishnan et al. is focused on the simulation of the digestive tract of salmon. The first part of the work is oriented to compare and standardize commercial enzymes and salmon-extracted enzymes based on their hydrolytic capacity on a purified protein substrate using pH-stat. The results demonstrated a positive correlation between them and also that the pH-stat method can be used to compare and standardize different sources of enzymes. The second part of the work develops a two stage *in vitro* hydrolysis model of several protein ingredients (a black soldier fly larvae meal and two experimental diets) and, after an alkaline step, the digested products were recovered for evaluating the amino acid solubility as well as to assess the correlation with the *in vivo* protein digestibility. The work demonstrated that results obtained when using salmon enzymes correlated better with the *in vivo* true protein digestibility.

A further two papers are oriented to evaluate the potential beneficial effects of different bioactive compounds obtained from vegetable sources in the metabolism of fish using different *in vitro* approaches as a part of the studies. The work by Zou et al. describes how pomelo fruitlets can be used to attenuate hepatic lipid accumulation in hybrid groupers. The authors prepared an active extract of the NaCl-soluble polysaccharide YZ-0.5A and characterized its molecular weight, monosaccharide composition, and chemical structure. Afterward, the polysaccharide was included at different doses from 150 to 1,200 mg/kg in diets for hybrid grouper. The results evidenced that the addition of 300**–**600 mg/kg of YZ0.5-A to feed significantly reduced lipid deposition in fish. In this case, the *in vitro* experiment consisted of the use of a culture of hepatocytes to investigate the YZ0.5-A concentration that may negatively affect hepatocyte viability. The *in vitro* experiments indicated that YZ0.5-A may alleviate unwanted lipid accumulation in fish liver and further inhibit oxidative stress and cell apoptosis.

On the other hand, the paper by Xia et al. uses the *in-vitro* culture of intestinal microbiota to demonstrate that berberine, the active ingredient of the Chinese herbal medicine *Coptis chinensis* that is widely used for the treatment of diabetes, is able to regulate glucose metabolism in largemouth bass by modulating intestinal microbiota. After feeding fish with a diet containing 1 g/kg diet of berberine for a 50-day period, the authors made *in-vitro* cultures of intestinal microbiota. The results revealed that consumption of berberine significantly increased the number of culturable bacteria and altered the gut microbial composition. The authors hypothesized that the blood glucose lowering effect that they found in live fish may be related to the regulation of intestinal microbiota composition.

Finally, the paper by Verdile et al. introduces a two-step approach to using *in vitro* techniques to simulate both digestion and intestinal absorption in rainbow trout. The work describes the use of different fish intestinal cell-based organotypic platforms and how prolonged exposure to a diet rich in fish meal may modulate the degree of cell differentiation. The authors used two cell lines derived from either the proximal and distal intestine cultured on three different platforms that were exposed for 21 days to increasing concentrations of feed pellets digested *in vitro* by gastric and intestinal enzymes. At the end of culture, epithelial cells formed multilayers irrespective of cell line or platforms if exposed to the products of the digesta but the controls did not. Overall, the three platforms reacted differently to prolonged exposure to the products of *in vitro* digestion: one of them showed no reaction, the second generated overly sensitive reactions, and the third reacted in a dose-dependent manner generating more physiological results. The results of the study point to the suitability of using intestinal organotypic platforms to perform functional studies aimed at predicting the intestinal permeability and/or absorption of nutrients and compounds present in novel feeds through the barrier system.

The above detailed are only some examples of how *in vitro* assays based on the use of fish enzymes and tissues can be applied to provide insights into fish nutrition and metabolism, becoming highly valuable experimental tools that reduce the use of experimental animals and help to get a better understanding of the biological mechanisms underlying the responses observed *in vivo.*

